# Epidemiological characteristics of 778 patients who underwent surgical drainage of chronic subdural hematomas in Brasília, Brazil

**DOI:** 10.1186/1471-2482-13-5

**Published:** 2013-03-01

**Authors:** Emerson B Sousa, Laise FS Brandão, Cléciton B Tavares, Igor BC Borges, Nelson G Freire Neto, Iruena M Kessler

**Affiliations:** 1 Service of Neurosurgery at the Hospital de Base do Distrito Federal, Brasília, Brazil; 2 Department of Medicine, University of Brasília, Brasília, Brazil; 3 Department of Pós-graduação em Gerontologia, Universidade Católica de Brasília, Brasília, Brazil

**Keywords:** Chronic subdural hematoma, Outcome, Surgery, Recurrence, Burr hole, Craniotomy, Epidemiology, Brazil

## Abstract

**Background:**

Chronic subdural hematomas (CSDHs) are common in neurosurgical practice. There are no publications that report large series of the epidemiological characteristics of this pathology in Brazil. The purpose is to describe a large series of surgical cases and analyze the epidemiological and clinical characteristics.

**Methods:**

We retrospectively analyzed patients with CSDH admitted into Neurosurgical Services at the Hospital de Base do Distrito Federal, Brasília, Brazil from 2006 to 2011. Age, sex, clinical feature, etiology, surgical procedure, side, clinical outcome, and recurrence were reviewed. Statistical tests were used to analyze data, and P < 0.05 was considered statistically significant.

**Results:**

The series included 778 patients. There were 643 (82.6%) male patients with a mean age of 64.3 ± 15.9 (range, 14–93) years. The principal symptom was headache (58.9%). The most frequent origin was a fall (282 cases, 36.2%), but the origin remained unclear in 281 (36.1%) patients. Mild head injury occurred in 540 (69.4%) cases. Burr holes with drainage were used as the surgical procedure in 96.5% patients, and 687 (88.3%) patients had a positive outcome. Mortality was 0%. Recurrence was observed in 42 cases.

**Conclusions:**

The occurrence of CSDHs is more common in elderly men. Treatment with burr holes and drainage is a simple and safe method for treatment. In our experience, CSDH presents decreased morbidity and mortality.

## Background

A chronic subdural hematoma (CSDH) is a slowly growing encapsulated collection of blood and its breakdown products located between the dura mater and the arachnoid. A CSDH is the result of tearing in the bridging veins, usually caused by minor trauma, and its risk factors include atrophy and coagulopathy in the brain [1,2]. Radiologically, a CSDH has been defined, based on the density discovered in the computed tomography (CT) scan, as a hypodense subdural hematoma, compared with parenchyma, and it presents 21 days after trauma [1].

A CSDH is one of the most common conditions encountered in neurosurgical practice. The common occurrence of CSDH in older patients causes a variety of diagnostic and therapeutic challenges because of frequently described nonspecific symptoms and accompanying diseases [1]. The incidence of CSDH increases greatly with age, and its occurrence ranges from approximately 3.4 per 100,000 in patients younger than 65 years of age to 8–58 per 100,000 in those older than 65 years [2,3].

The, presentation, management, and prognosis of CSDHs are well-documented, but there are few publications that report the epidemiological characteristics of this pathology in Brazilian neurosurgery [4-6]. A series of surgical CSDH cases from our institution will be presented, and the epidemiological and clinical characteristics, causes, surgical results, and recurrence rate will be described.

## Methods

**Figure 1:**
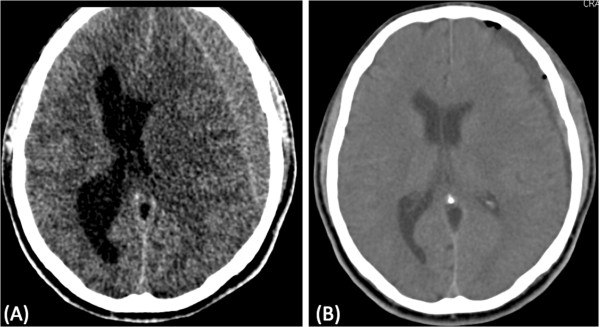
**Computerized tomography scans (CT). **(**A**) chronic subdural hematoma; (**B**) CT-scan 24 h after surgery.

We retrospectively analyzed all patients admitted to Neurosurgical Services at the Hospital de Base do Distrito Federal, Brasília, Brazil with a CSDH between January 2006 and December 2011. In all cases, the diagnosis of CSDH was made using the cranial CT scan of the patient performed before the surgical procedure that showed a crescent-shaped hypodense or isodense hemispheric collection of blood layered over the cerebral convexity, independent of knowledge of an occurrence of a traumatic injury (Figure 1). A surgical evacuation was performed when the hematoma’s thickness was larger than 10 mm, when focal symptoms were present, or when there were significant changes in neurologic status in patients with CSDH of any thickness. Anticoagulant/antithrombotic drugs were stopped if used by a patient. After a normal international normalized ration was confirmed, the surgery was performed. In case of an emergency procedure and coagulopathy, anticoagulant status was reversed with vitamin K and fresh frozen plasma.

The neurosurgical staff on duty selected the type of surgical procedure (burr hole with closed-system drainage or craniotomy). The surgery was performed under general anesthesia by creating one burr hole of approximately 10 mm in diameter in the side of the location of the hematoma. After exposing the dura mater and the outer membrane of the hematoma, the collection of blood was evacuated under its own tension, and irrigation was performed using physiological saline solution until clear fluid came out. When the subdural space allowed, a closed subdural drainage system, without any negative pressure, was placed and subcutaneously tunneled for at least 5 cm. Subdural drainage was continued up to 48 h after surgery, during which time, the patient was supine in bed. During the craniotomy, a piece of bone was removed and the largest portion of the brain was exposed. After exposing the dura mater and the outer membrane of the hematoma, only the outer membrane was removed. After evacuation of the hematoma, the piece of bone was replaced and fixed to the skull. Surgical hemostasis was performed with monopolar/bipolar cautery and oxidized regenerated cellulose (Surgicel ^R^, Johnson & Johnson, Arlington, TX, USA).

After the surgery, the patients routinely received seizure prophylaxis with 15 mg/Kg of phenytoin by slow IV, followed by 100 mg IV every 8/8 h, and prophylactic antibiotic with cefazolin 1 g IV every 8/8 h for 48 h. The patients stayed in the post-anesthesia recovery room and in the neurosurgical ward. A cranial CT was only performed in patients with clinical complications. Subcutaneous injection of 40 mg of enoxaparin was used after 24 h of surgical procedures for prophylaxis against deep vein thrombosis during the patient’s stay in hospital.

We reviewed age, sex, the presenting signs and symptoms, Glasgow Coma Scale (GCS), origin, type of surgical procedure, side of the skull in which the hematoma occurred, days of hospitalization, Glasgow Outcome Scale (GOS) at discharge, and the recurrence of CSDH for each patient. CSDH recurrence was determined when the clinical status did not improve after the operation or new neurological symptoms occurred with re-accumulation of a subdural blood collection, as seen on a CT scan. There was no follow-up of patients after discharge from hospital.

All data were analyzed using the commercially available statistical software package SPSS 20.0 (SPSS^®^, Chicago, IL, USA). Continuous distributed variables were separately compared using the student’s *t*-test, and they were reported as mean ± standard deviations (SD). Proportions were compared with chi-square and Fisher’s exact test, and they were presented as percentages. P < 0.05 was considered statistically significant. The study protocol was approved by the institute’s committee on human research (Comitê de Ética em Pesquisa da Secretaria de Saúde do Distrito Federal, protocol 138/2012).

## Results

**Figure 2:**
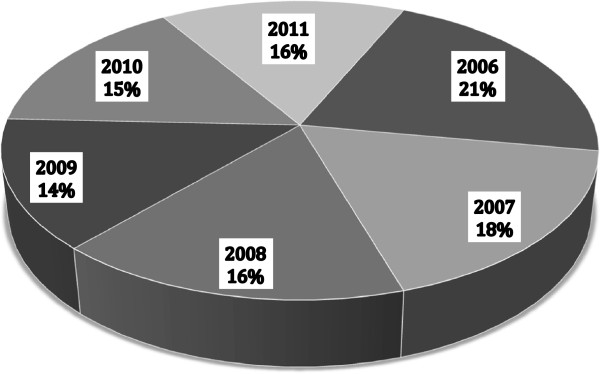
Annual incidence of 778 patients with CSDH.

**Figure 3:**
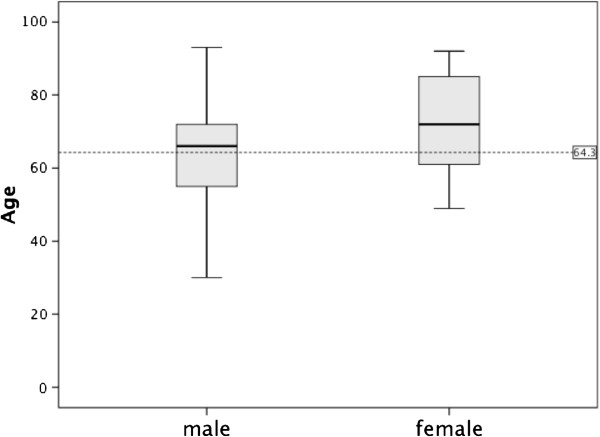
**Box-and-whisker plot of age as a function of sex of patients with CSDH. **The lines demarcate the median and inter-quartile range, and the whiskers indicate the upper and lower limits of the data. The dashed line demarcates the mean age (in years) of 778 patients who underwent CSDH operations in Neurosurgical Services at the Hospital de Base do Distrito Federal, Brasília, Brazil.

**Figure 4:**
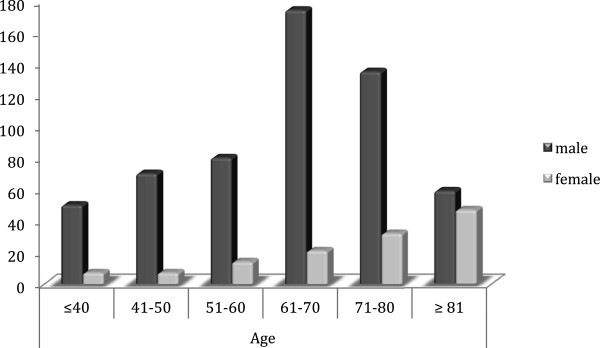
Bar graph of sex as a function of age for 778 patients with CSDH.

The sample consisted of 778 patients. The annual incidence of CSDH is illustrated in Figure 2. Of the cases, 643 were male, and 135 were female (ratio 4.8:1). The age range was 14–93 years, the mean age for all patients was 64.3 ± 15.9 years, and the mean ages for male and female patients were 63.0 ± 14.9 and 70.0 ± 18.8 years, respectively (P < 0.001; Figure 3). An age 65 years or older was associated with a more frequent incidence of CSDH: 56.8% (n = 442) of the patients were older than 65 years, whereas 43.2% (n = 336) were younger. In patients younger than 65 years, 87.5% were male, and 12.5% were female; in patients 65 years of age and older, 79% were men, and 21% were women (P < 0.005). Table 1 presents the main characteristics of the population in study, and Figure 4 shows the distribution of sex based on age.

**Table 1:** Characteristics of 778 patients with CSDH

**Sex**	
Male (%)	643 (82.6%)
Female (%)	135 (17.4%)
**Age in years (mean ± SD) and range**	64.3 ± 15.9 (14–93)
<65 years (%)	336 (43.2%)
≥65 years (%)	442 (56.8%)
**Median (IQR) GCS on admission**	14 (13–15)
**GCS (%)**	
15-14	540 (69.4%)
13-9	192 (24.7%)
8-3	30 (3.9%)
**Convexity hematoma (%)**	
Left	293 (37.7%)
Right	311 (40.0%)
Bilateral	174 (22.3%)
**Origin**	
Fall	282 (36.2%)
Traffic accident	74 (9.5%)
Aggression	35 (4.5%)
Other accidents	79 (10.1%)
Coagulopathy or anticoagulant/ antiaggregant therapy	27 (3.5%)
Unclear	281 (36.1%)

Values shown are mean ± standard deviations (SD), unless otherwise specified, and percentages, where indicated. *GCS *= Glasgow coma scale; *IQR *= Interquartile range.

The origin of the CSDH was determined in 497 patients (63.9%) and remained unclear in 281 patients (36.1%). Of the determined cases, 470 had suffered a previous head injury, and 27 patients had coagulopathy or used an anticoagulant/antiaggregant therapy without a history of trauma associated with the treatment. The causes of the head injuries were a fall (282 cases), traffic accident (74 cases), aggression (35 cases), or other accidents (79 cases). Falls represented 60.7% (82 of 135 cases) of the causes of CSDH in female patients, but they accounted for only 31.1% (200 of 643 cases) in the male patients (P < 0.001). Falls also represented the most frequent origin of trauma in patients aged 65 years or older (154 cases) and in patients younger than 65 years (96 cases; P > 0.05). Traffic accidents and aggression were significantly more frequent in patients younger than 65 years of age (P < 0.001 and P < 0.01, respectively).

The most frequent symptom of CSDH was headache (458 cases; 58.9%), which was followed by altered behavior (272 cases; 35%) and hemiparesis (271 cases; 34.8%). The frequency of the presenting symptoms is shown in Table 2. The leading symptom for the age and the sex was headache, hemiparesis and behavioral disturbance. Altered behavior was significantly more frequent in patients with 65 years of age or older than in younger patients (38.7% versus 22%; P < 0.001). Mild head injury (GCS 15–14 points) occurred in 540 patients (69.4%), moderate head injury (GCS 13–9 points) occurred in 192 patients (24.7%), and severe head injury (GCS 8–3 points) occurred in 30 patients (3.9%).

**Table 2:** Symptoms of 778 patients with CSDH^*^


**Headache**	458 (58.9%)
**Hemiparesis**	271 (34.8%)
**Cognitive disturbances**	176 (22.6%)
**Altered behavior**	272 (35.0%)
**Seizure**	20 (2.6%)

Values are n (%).*More than one symptom per patient is possible.

The postoperative results are shown in Table 3. The CSDH was on the right side of the brain in 293 patients (37.7%), the left side in 311 (40.0%), and bilateral in the remaining 174 (22.3%) cases. Bilateral CSDH occurred significantly more often in younger patients than in older patients (26.5% versus 19.2%; P < 0.05) and in male patients more than female patients (26.3% versus 5.2%; P < 0.001). The surgical procedures undertaken in patients with CSDH were burr holes with drainage in 96.5% of cases (751 patients) and craniotomy in 3.5% of cases (27 patients). A total of 42 (5.4%) patients required further surgery to remove a recurring CSDH, and one case underwent a third operation. The average time of the second intervention was approximately 4 weeks with a 71.4% recurrence rate within 3 months.

**Table 3:** Postoperative results of 778 patients with CSDH

**Surgical procedures**	
Burr holes with drain	751 (96.5%)
Craniotomy	27 (3.5%)
**Recurrence**	42 (5.4%)
Within 3 months	30 (71.4%)
GOS, median (IQR) on admission*	5 (5–5)
5	687 (88.3%)
4	56 (7.2%)
3	35 (4.5%)
**Days hospitalized (mean ± SD)**	6.53 ± 7.7

Values shown are mean ± standard deviations (SD), unless otherwise specified, and percentages, where indicated. *GOS *= Glasgow outcome scale (*5 *= Good recovery; *4 *= Moderate disability with the ability to live independently; *3* = Severe disability, unable to live independently; *2 *= Vegetative state; *1* = Dead); *IQR *= Interquartile range. *There were no patients with 1 or 2 GOS scores.

Of the patients, 687 (88.3%) had a positive outcome (GOS 5). Patients with severe brain injuries upon admission had poor outcomes (GOS 3–2) compared with patients with mild and moderate brain injuries (50.0%, 0.9%, and 7.8%, respectively; P < 0.001). Postoperative mortality was 0%.

**Figure 5:**
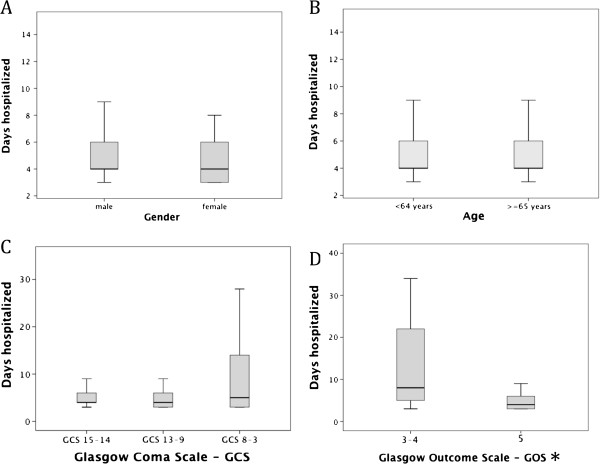
**Box-and-whisker plot of days hospitalized for A) Gender B) Age C) GSC D) GOS of 778 patients with CSDH. **The lines demarcate the median and interquartile range, and the whiskers indicate the upper and lower limits of the data of 778 patients who underwent CSDH operations in Neurosurgical Services at the Hospital de Base do Distrito Federal, Brasília, Brazil. GOS = Glasgow outcome scale (5 = Good recovery; 4 = Moderate disability with the ability to live independently; 3 = Severe disability, unable to live independently; 2 = Vegetative state; 1 = Dead); GCS = Glasgow coma scale. *There were no patients with GOS scores of 1 or 2.

The mean stay in the Department of Neurosurgery was 6.53 ± 7.7 days, ranging from 3 to 58 days (median, 4 days); 82% were discharged within 1 week, 9.8% within 2 weeks, and the remaining patients after 2 weeks. The average number of days of hospitalization for patients with GCS 8–3 (10.6 ± 9.8 days) was statistically greater than patients with GCS 15–14 and GCS 13–9 (6.3 ± 8.0 and 6.85 ± 7.3, respectively; P < 0.02). Patients with poor outcomes (GOS 3–2) had a greater mean of days hospitalized than patients with good recoveries (24.5 ± 18.5 and 5.7 ± 5.6 days, respectively; P < 0.001; Figure 5).

## Discussion

In 1657, J. J. Wepfer was the first to describe a CSDH, and in 1857, Virchow detailed the pathophysiology of this hematoma, naming it pachymeningitis haemorrhagica interna [1,7]. Many authors have since demonstrated that lesions are easily treated with minimal morbidity or mortality.

CSDH occur in the dural border cell layer, located between the dura mater and the arachnoid. The dissection of these cell layers creates a subdural cavity [8]. Patients with extensive brain atrophy (elderly and alcoholics) or conditions resulting in intracranial hypotension (ventriculoperitoneal shunt) are vulnerable to developing CSDH [1,2]. Traversing veins are being increasingly stretched by the shrinking brain until only a minor additional force is sufficient to cause the rupture of the bridging veins and create the hematoma. This is followed by fibrin deposition, organization, enzymatic fibrinolysis, and liquefaction of the clot [1,2,9-11]. An inflammatory reaction occurs, and neomembranes (inner or visceral and outer or parietal membranes) are formatted with the growth of neocapillaries and enzymatic hyperfibrinolysis. CSDH tend to gradually enlarge because repeated micro-hemorrhage may lead to clinical signs and symptoms of increased intracranial pressure or compression brain structures [2,9-12].

The elderly and male preponderance of CSDH has already been described [1-3,13-15]. In the elderly, the brain weighs approximately 200 g less, which leads to an approximate 11% increase in extracerebral volume, allowing for greater movement of the brain [16]. Other factors explain why older people have a predisposition for trivial trauma. One rationale for male dominance could be that men generally have a greater exposure to injuries [15]. Studies show that male predominance diminishes with age [3,9,15]. This tendency to match the ratio between the sexes can be seen in our results also (Figure 3), and it can explain the greater life expectancy of women [3].

The history of trauma was acquired in 60.4% of patients. Among these, 282 (60%) had a history of a fall (a trivial trauma). This is comparable to many studies that show an average of 56 to 77% of CSDH patients with a history of a fall [13-15,17]. In our results, traffic accidents and aggression were most frequent in patients with younger than 65 years of age, as well as in males; also, younger people generally have a greater exposure to injuries [15].

Chronic anticoagulation/antiaggregant therapy uses are also at increased risks for CSDH. Although the process is incompletely established, it has been suggested that asymptomatic “microbleeds” permit the development of a symptomatic hemorrhage [18]. These drugs, which are used in a large proportion of the elderly population, may add to the risk of CSDH by as much as 42.5 times [19]. Some studies report that patients initially taking anticoagulant medications have more risk of recurrence and longer stays in the hospital [2,3,7,13,15,20]. Our results show only 3.5% of all CSDH patients admitted were chronically anticoagulated/antiaggregated or had coagulopathy history. In one study, 41% of all CSDH patients admitted to a neurosurgical department in Switzerland were chronically anticoagulated [15]. Our data may be explained by incomplete medical records and by deficiencies in Brazilian public health in diagnosing pathologies requiring the chronic use of these drugs, as well as deficient distribution of these medicaments to the population.

Headache, hemiparesis, and behavioral disturbance were frequent symptoms in our study and in related articles [3,5,13]. In our series, behavioral disturbances were more frequent in patients 65 years of age or older compared with younger patients who frequently presented with increased intracranial pressure (headache). Gelabert-González et al. [3] and Asghar et al. [7] reported that the most common clinical characteristics in elderly patients were mental disorders.

Accepted management of CSDH (burr hole with or without closed-system drainage, twist-drill craniostomy, or craniotomy) is accompanied by recurrence rates of 4 to 26% [2,3,7,13,15]. In our series, 96.5% of the surgical procedures undertaken in patients with CSDH were burr holes with closed-system drainage, and only 5.4% of patients experienced a recurrence. Craniotomy is the most invasive, encompassing the longest operating time and the greatest blood loss and remains with option in calcified or CSDH with numerous thick membranes [2]. Twist-drill craniostomy can be performed at the bedside in patients with multiple medical co-morbidities; however, there is a theoretical increased risk of contamination [21].

The most frequently used technique is burr-hole craniostomy with or without drainage [2]. The debate regarding the role of a drainage system in the surgical management of this pathology is ongoing. According to some reports, the installation of a drainage system helps brain expansion, decreasing the chance of recurrence [10]. However, the reported complications of a drainage system are significant, including hemorrhage, seizure induction, and infection [2,3,10,11,20]. Thus far, doubt persists about whether the installation of a drainage system is safer and more useful than irrigation without leaving a drainage system in place. Perhaps another explanation for the low recurrence rate of CSDH in our study is the lack of follow-up data of patients.

Positive functional results (score of 5 in GOS) have been shown in 72-93% of patients in several reports with the various types of surgical procedures, including twist-drill craniostomy, burr-hole craniostomy, and craniotomy [21,22]. Our patients also conform to this trend (88.3%).

The mortality rate varies in a recent series from 0 to 13% [3,22], and in our series, the mortality rate was 0%. Age, systemic complication, coagulopathy, and poor preoperative neurological state are contributory causes of postoperative death, functional outcome, and length of stay in the hospital [2,3,10,11,20]. This was evident in our study, as well.

Among the limitations of our study are the lack of personal history data, the tomographic imaging of the hematoma, and complications in patients who underwent operations without complete medical records. These limitations can be discussed in future research.

## Conclusion

Chronic subdural hematomas are common neurosurgical problems associated with significant morbidity and mortality. In our series, headache and behavioral disturbance were the most frequent signs of CSDHs in elderly patients, and headache was the most frequent symptom in younger patients. A burr hole with closed-system drainage is a simple, safe, and efficient method for the treatment of CSDH, and it has a low occurrence of complications. In our experience, the worst prognostic factor for the outcome of CSDH in patients was the neurological condition at the time of surgery.

## Abbreviations

CSDH: Chronic subdural hematoma; CT: Computerized tomography scan; GCS: Glasgow coma scale; GOS: Glasgow outcome scale; SD: Standard deviations; IQR: Interquartile range.

## Competing interests

The authors declare that they have no competing interests.

## Authors’ contributions

EBS and LFSB were the key authors for the conception, design, coordination, and drafting of the manuscript, as well as the analysis and interpretation of the data. CBT and IBCB participated in the design and interpretation of the data and helped in drafting the manuscript. NGFN and IMK contributed substantively by revising the manuscript critically for intellectual content and participating in the interpretation of data and the revision of the manuscript. All authors read and approved the final manuscript.

## Pre-publication history

The pre-publication history for this paper can be accessed here:

http://www.biomedcentral.com/1471-2482/13/5/prepub
